# Synergistic enhancement of 5-fluorouracil cytotoxicity by deoxyuridine analogs in cancer cells

**DOI:** 10.18632/oncoscience.125

**Published:** 2015-02-09

**Authors:** Yoshihiro Matsumoto, Victoria Rodriguez, Tracy A. Whitford, Neil Beeharry, Hiroshi Ide, Alan E. Tomkinson

**Affiliations:** ^1^ Department of Internal Medicine and University of New Mexico Cancer Center, University of New Mexico, Albuquerque, New Mexico, USA; ^2^ Fox Chase Cancer Center, Philadelphia, Pennsylvania, USA; ^3^ Department of Biological Sciences, East Stroudsburg University, East Stroudsburg, Pennsylvania, USA; ^4^ Department of Mathematical and Life Sciences, Hiroshima University, Higashi-Hiroshima JAPAN; ^5^ Howard Hughes Medical Institute Student Scientist, Fox Chase Cancer Center, Philadelphia, Pennsylvania, USA; ^6^ Department of Communication Sciences and Disorders, Temple University, Philadelphia, Pennsylvania; ^7^ LAM Therapeutics, Guilford, Connecticut

**Keywords:** Synergy, Cytotoxicity, 5-Fluorouracil, Deoxyuridine Analog

## Abstract

5-Fluorouracil (FU) is a halogenated nucleobase analog that is widely used in chemotherapy. Here we show that 5-hydroxymethyl-2′-deoxyuridine (hmUdR) synergistically enhances the activity of FU in cell lines derived from solid tumors but not normal tissues. While the cytotoxicity of FU and hmUdR was not directly related to the amount of the modified bases incorporated into cellular DNA, incubation with this combination resulted in dramatic increase in the number of single strand breaks in replicating cancer cells, leading to NAD-depletion as consequence of poly(ADP-ribose) synthesis and S phase arrest. Cell death resulting from the base/nucleoside combination did not occur by apoptosis, autophagy or necroptosis. Instead, the cells die via necrosis as a result of NAD depletion. The FU-related nucleoside analog, 5-fluoro-2′-deoxyuridine, also displayed synergy with hmUdR, whereas hmUdR could not be replaced by 5-hydroxymethyluracil. Among other 5-modified deoxyuridine analogs tested, 5-formyl-2′-deoxyuridine and, to a lesser extent, 5-hydroxy-2′-deoxyuridine, also acted synergistically with FU, whereas 5-hydroxyethyl-2′-deoxyuridine did not. Together, our results have revealed an unexpected synergistic interaction between deoxyuridine analogs and FU in a cancer cell-specific manner, and suggest that these novel base/nucleoside combinations could be developed into improved FU-based chemotherapies.

## INTRODUCTION

Since its first rational development in 1957, 5-fluorouracil (FU) has been widely used as a chemotherapy reagent for various types of cancers, including colorectal, breast and pancreatic cancers [1]. FU is an antimetabolite that exerts its cytotoxic effect via several different mechanisms. These include reducing dTTP levels by inhibition of thymidylate synthase, misincorporation of both dUTP and FdUTP during DNA replication and repair of misincorporated dUTP and FdUTP, misincorporation of FUTP into RNA and disruption of several aspects of RNA metabolism. Through its long history, the mechanism of action of FU has been studied extensively, and a number of derivatives and combination therapies with other types of therapeutics have been developed to improve its effectiveness [2]. Nevertheless these combination therapies often increase the risk of severe side effects limiting clinical application, and many tumor types exhibit a low response rate and/or rapidly acquire resistance [3].

5-Hydroxymethyl-2′-deoxyuridine (hmUdR) is a deoxyuridine analog, which can be formed by oxidation of thymine in cellular DNA exposed to ionizing radiation [4,5]. When added to culture medium, hmUdR is incorporated into cellular DNA, causing cytotoxicity in tumor cells [6-9]. Interestingly, it has been reported that hmUdR synergistically enhances the growth inhibitory activity of 1-β-D-arabinofuranosylcytosine (Ara-C) by increasing the incorporation of the modified nucleoside into cellular DNA [10]. While examining the cytotoxicity of a number of base adducts generated by ionizing radiation, we found that a combination of FU and hmUdR inhibited cell proliferation much more potently than either compound alone. Here we demonstrate that hmUdR and other deoxyuridine analogs synergistically enhance the cytotoxicity of FU in cancer but not normal cells by dramatically increasing the number of single strand breaks.

## RESULTS

### The combination of FU and hmUdR has a much greater effect on cell survival than either agent alone

**Figure 1:**
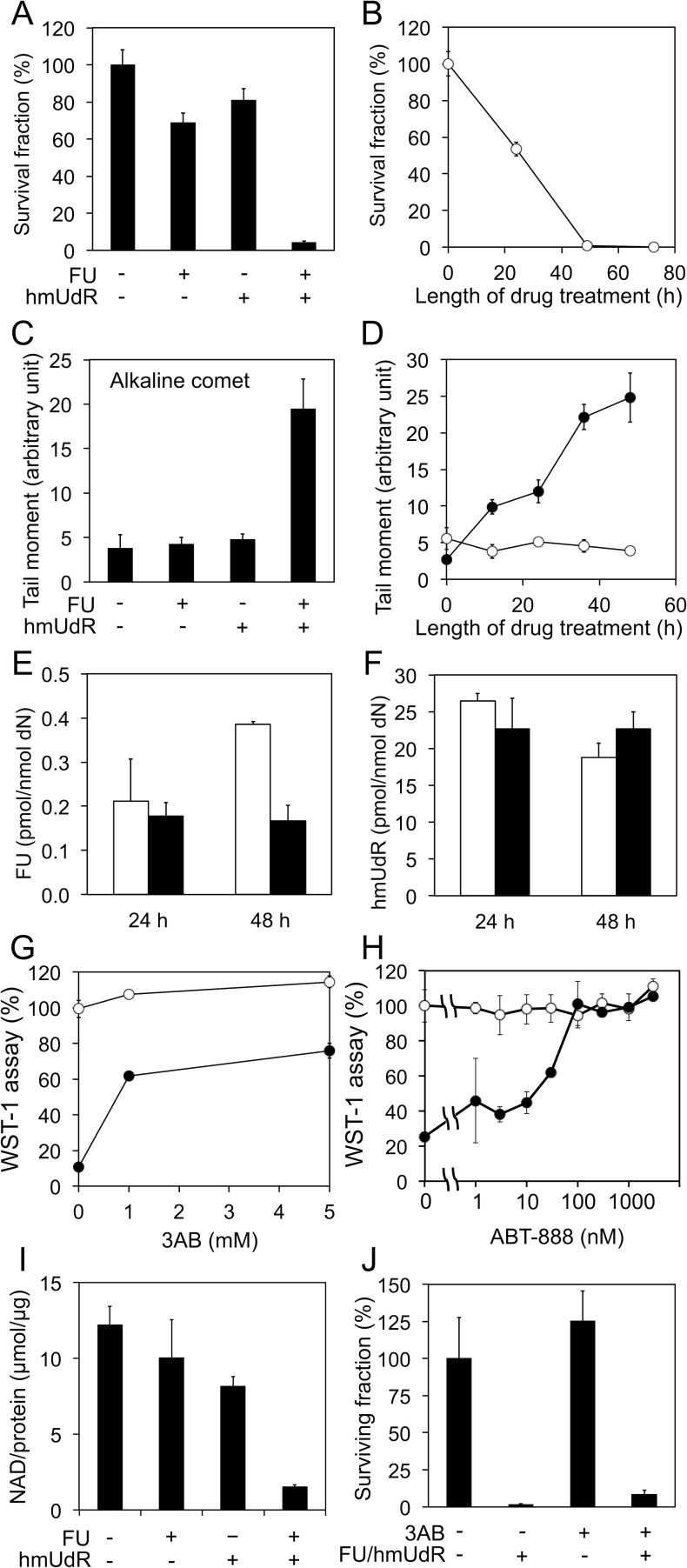
Properties of the synergistic toxicity by FU and hmUdR (A) Colony formation assays of HT-29 cells treated for 48 h with or without 0.5 μM FU and/or 5 μM hmUdR. (B) Time course of effects of FU and hmUdR in colony formation assay. (C) Alkaline comet assays for detection of single-strand breaks (SSBs) in HT-29 cells treated for 48 h with indicated combinations of 0.5 μM FU and 5 μM hmUdR. (D) Time course of SSB formation. The SSB formation was quantitated in HT-29 cells treated with (•) or without (○) 0.5 μM FU and 5 μM hmUdR. (E) Incorporation of FU into HT-29 cellular DNA. Incorporation of tritium-labeled FU (0.5 μM in the medium) was measured in the absence (□) or the presence (■) of 5 μM hmUdR and presented as picomoles per nanomoles of deoxynucleosides. (F) Incorporation of hmUdR into HT-29 cellular DNA. Incorporation of tritium-labeled hmUdR (5 μM in the medium) was measured in the absence (□) or the presence (■) of 0.5 μM FU and presented as picomoles per nanomoles of deoxynucleosides. (G) Effects of 3-aminobenzamide (3AB), a broad PARP inhibitor on the cytotoxicity by FU and hmUdR. 3AB was titrated for its effect on the HT-29 cell growth in the absence (○) or the presence (•) of 0.5 μM FU and 5 μM hmUdR. 3AB was added to the medium simultaneously with FU and hmUdR. The cell growth was measured by WST-1 assay. (H) Effects of ABT-888, a specific inhibitor for PARP1 and PARP2, on the cytotoxicity by FU and hmUdR. ABT-888 was titrated for its effect on the HT-29 cell growth in the absence (○) or the presence (•) of 1 μM FU and 10 μM hmUdR. ABT-888 was added to the medium simultaneously with FU and hmUdR. The cell growth was measured by WST-1 assay. (I) Effect of FU and hmUdR on cellular NAD levels. The quantities of NAD in cell extracts were normalized with the protein concentrations of the extracts. (J) Survival fractions of HT-29 cells treated with drugs in the presence of 3AB for 72 h. After replating without drugs, the cells were allowed to grow for 6 days and their nucleic acids were quantitated by CyQUANT kit. Data in panels A-J are from triplicate experiments and plotted with standard deviations.

Although nucleoside/base analogs, such as FU and gemcitabine, have been used as cancer therapeutics for many years, there have been relatively few efforts to examine the activity of combinations of nucleoside analogs. In initial studies, we focused on hmUdR, a derivative of thymidine generated by ionizing radiation that is cytotoxic when added to cancer cells cultured *in vitro* [6-9]. The combination of FU and hmUdR markedly reduced colony formation in p53 mutant colorectal adenocarcinoma HT-29 cells compared with either compound alone, suggesting that these compounds together synergistically increase cytotoxicity (Figure 1A). Colony formation was reduced by about 50% after incubation with FU and hmUdR for 24 h and by more than 95% after incubation for 48 h (Figure 1B).

### Effects of FU and hmUdR on the integrity of genomic DNA

To gain insights into the mechanisms underlying the apparent synergistic activity of FU and hmUdR, we examined genome integrity using single cell gel electrophoresis (comet) assays under alkaline conditions. While incubation with either FU or hmUdR did not significantly increase the number of single-strand breaks, there was a dramatic increase in the number of DNA single strand breaks when HT-29 cells were incubated with both FU and hmUdR (Figure 1C). As expected, the number of strand breaks increased with increasing time of incubation with the combination of FU and hmUdR (Figure 1D). In contrast, the number of double strand breaks measured in a neutral comet assay increased when cells were incubated with hmUdR whereas FU has no significant effect on DNA double strand break formation in either absence or presence of hmUdR (Supplementary Figure 1). Thus we conclude that the increase in the number of single- but not double-strand breaks in genomic DNA correlates with the enhanced cytotoxicity of the FU and hmUdR combination.

To determine whether either FU or hmUdR modulates the incorporation of the other compound into cellular DNA, we measured the incorporation of tritium-labeled derivatives of FU and hmUdR in the absence or presence of the other compound. As shown in Figure 1E and F, incorporation of FU was not stimulated by the presence of hmUdR nor vice versa. The incorporation of hmUdR estimated here appears much higher than the incorporation of hmUdR previously measured in U2OS cells [11]. This is probably because HT-29 cells have extremely weak activity for excision of hmU (Supplementary Figure 2). It should be noted that incorporation of FU at 48 h was decreased in the presence of hmUdR. While this may reflect increased cell death, it is clear that the increased number of single-strand breaks observed in cells incubated with the combination of FU and hmUdR is not simply due to increased FU or hmUdR incorporation into cellular DNA.

### Hyperactivation of poly (ADP-ribose) polymerase 1 and NAD depletion in cells incubated with the combination of FU and hmUdR

The poly(ADP-ribose) polymerase, PARP1, plays a major role in the cellular response to single strand breaks [12]. This enzyme binds to and is activated by single strand breaks, resulting in the synthesis of poly (ADP-ribose) chains on PARP1 itself and other proteins in the vicinity. In accord with our results showing that co-incubation with FU and hmUdR results in a synergistic increase in the number of single-strand breaks, the levels of poly (ADP-ribose) were much higher in cells treated with FU and hmUdR compared with either compound alone (Supplementary Figure 3). Since NAD is the substrate for poly (ADP-ribose) synthesis, it is likely that NAD levels in cells treated with FU and hmUdR will be reduced. To test this idea, we measured the activity of the mitochondrial succinate-tetrazolium reductase complex that is dependent upon cellular NAD(P)/NAD(P)H levels using the WST-1 assay. As expected, incubation of cells with FU and hmUdR resulted in reduced succinate-tetrazolium reductase activity (Figure 1G and H). This reduction in activity was partially corrected by the inhibition of poly (ADP-ribose) synthesis using PARP inhibitors, either 3-aminobenzamide (3AB, Figure 1G) or ABT-888 (Figure 1H). Furthermore we directly measured the cellular levels of NAD in the cells treated with FU and hmUdR, and observed that the combination treatment with these compounds drastically decreased the NAD level (Figure 1I). To examine whether PARP inhibition can restore cell proliferation and viability, we examined the effect of FU and hmUdR on cell proliferation by using a CyQUANT assay that measures cellular nucleic acid (Figure 1J). In accord with the colony forming assays, the combination of FU and hmUdR dramatically reduced cell proliferation, and the PARP inhibitor, 3AB, did not rescue the effect of FU and hmUdR on cell proliferation.

### Effects of FU and hmUdR on cell cycle progression

**Figure 2:**
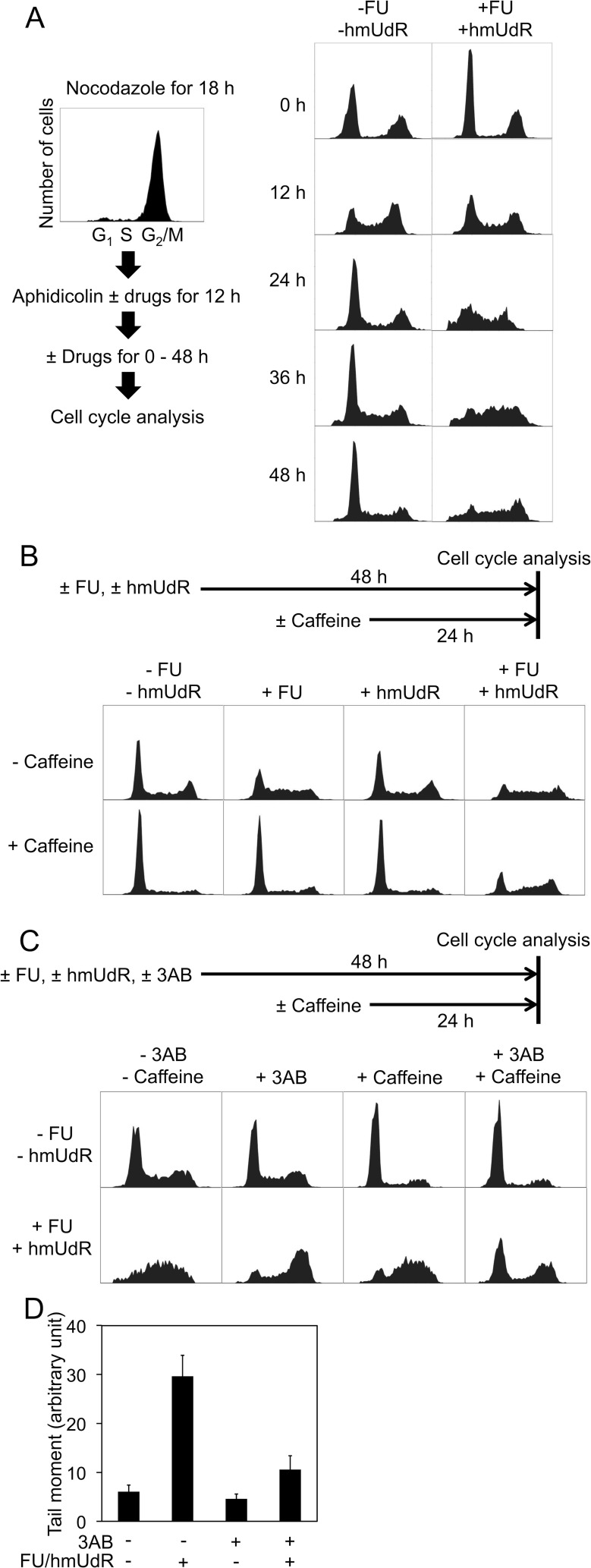
Cell cycle analyses of HT-29 cells by flow cytometry (A) Time course of cell cycle distribution of synchronized cells treated with a combination of 0.5 μM FU and 5 μM hmUdR. HT-29 cells were synchronized at the G /S boundary by sequential pretreatment with nocodazole and aphidicolin as described in Materials and Methods. The time at which aphidicolin was removed is designated 0 h. When indicated, FU and hmUdR were added through aphidicolin treatment and subsequent incubation. (B) Effect of FU, hmUdR and caffeine on cell cycle distribution. Unsynchronized HT-29 cells were treated without or with 0.5 μM FU and 5 μM hmUdR for 48 h, and incubated in the absence or presence of 5 mM caffeine for the last 24 h. (C) Cell cycle analyses of unsynchronized HT-29 cells in the presence of 3AB and caffeine. (D) Alkaline comet assay of HT-29 cells treated for 48 h with drugs in the presence of 3AB. In both experiments, 0.5 μM FU, 5 μM hmUdR and 3 mM 3AB were added when indicated. Data in panel D are from triplicate experiments and plotted with standard deviations.

HT-29 cells were synchronized at the G /S boundary by sequential treatments with nocodazole and aphidicolin. FU and hmUdR were added to the medium during the aphidicolin treatment and then maintained after aphidicolin removal (Figure 2A). Although one third of the cell population remained in G /M phase after the aphidicolin treatment due to incomplete recovery from the nocodazole treatment, the majority of both treated (61%) and untreated cells (58%) were in the G_1_ phase and S phase cells were scarce (10% of untreated and 11% of treated cells). Following removal of aphidicolin and incubation for 12 h, 44% of untreated cells and 41% of treated cells were in S phase. By 24 h, the untreated cell population exhibited a normal cell cycle distribution with a major G_1_ population. In contrast, the majority of treated cells remained in S phase up to 48 h after the removal of aphidicolin. To confirm that these cells are trapped in S phase, we analyzed the frequency of cell division for approximately two cell-cycle periods by time-lapse video microscopy. When untreated cells were analyzed, the number of cell divisions observed per view field during the second 24 h period was 1.6 times (± 0.6) the number during the first 24 h period, indicating continued cell cycle progression. Similarly the cells treated with either 0.5 μM FU or 5 M hmUdR alone had ratios of 1.5 ± 0.3 and 1.4 ±0.2, respectively. In contrast, the cells treated with both FU and hmUdR divided much less frequently in the second 24 h of treatment, 0.5 times (± 0.3) the number observed during the first 24 h. Thus, co-incubation with FU and hmUdR results in cell cycle arrest mainly in the first S phase after the FU/hmUdR addition.

To further characterize this cell cycle arrest, we examined the effects of FU and hmUdR alone compared with their combination (Figure 2B). Treatment with FU alone caused cells to accumulate in S phase (52%), although to a lesser extent than after treatment with both FU and hmUdR (64%) whereas hmUdR alone did not change the cell cycle distribution. Interestingly, the S phase arrest induced by FU alone was abolished when cells were treated with caffeine, an ATM/ATR inhibitor, whereas the S phase arrest induced by the combination of FU and hmUdR was resistant to caffeine, indicating that the cell cycle arrest induced by the combination is mechanistically distinct from that induced by FU alone (Figure 2B).

To determine whether FU and hmUdR inhibit DNA replication in the absence of NAD depletion, we examined the effect of 3AB on the S phase arrest induced by FU and hmUdR (Figure 2C). Addition of 3AB simultaneously with FU and hmUdR enabled most cells to progress through S phase to G /M. We also observed by alkaline comet assay that the same treatment significantly decreased the number of strand breaks compared to the cells treated without 3AB (Figure 2D), suggesting that inhibition of PARP activation by 3AB not only enables cells to continue DNA replication but also to repair a significant fraction of, if not all, replication-dependent DNA damage caused by FU and hmUdR. The accumulation of G /M cells when incubated with 3AB in addition to FU and hmUdR suggests that residual replication-dependent DNA damage activates the G /M checkpoint. In support of this idea, the addition of caffeine partially released the G /M arrest, resulting in the emergence of G_1_ cells (Figure 2C).

### Mechanism of cell death induced by FU and hmUdR

**Figure 3:**
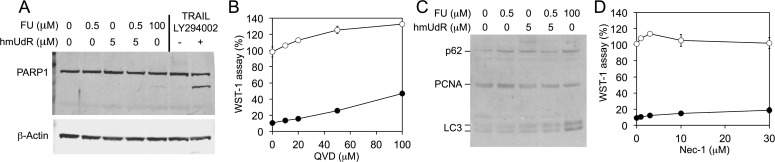
Characterization of the mechanism for cell death resulting from combined treatment with FU and hmUdR (A) Immunoblot detection of PARP1. PARP1 cleavage was examined in whole cell extracts of HT-29 cells treated for 72 h with indicated concentrations of FU and hmUdR. As a positive control for PARP1 cleavage, HT-29 cells were treated with 50 μM LY294002 for 1 h followed by 4 h treatment with 100 μg/ml TRAIL. β-Actin was a loading control. (B) Effects of an apoptosis inhibitor. A broad spectrum caspase inhibitor, QVD, were tested for their effects on the HT-29 cell growth in the absence (○) or the presence (•) of 0.5 μM FU and 5 μM hmUdR. QVD was added to the medium simultaneously with FU and hmUdR. The cell growth was measured by WST-1 assay. The slight increase in cell growth with 50 and 100 μM QVD was an effect of DMSO in which QVD was dissolved. (C) Immunoblot detection of autophagy-related proteins, p62 and LC3 (microtubule-associated protein 1 light chain 3). p62, LC3 and a loading control, PCNA, were detected in the whole cell extracts prepared by the same way as for panel A. Autophagy is expected to decrease p62 and increase the LC3 proteins. (D) Effects of a necroptosis inhibitor on the cytotoxicity by FU and hmUdR. Necrostatin-1 (Nec-1) was tested for their effects on the HT-29 cell growth in the absence (○) or the presence (•) of 0.5 μM FU and 5 μM hmUdR. Nec-1 was added to the medium simultaneously with FU and hmUdR. The cell growth was measured by WST-1 assay. Data in panels B and D are from triplicate experiments and plotted with standard deviations.

We sought to investigate the mechanism by which cells die following the combination treatment. In initial studies, we asked whether the combination of FU and hmUdR induced apoptosis. PARP1 cleavage, a characteristic of apoptosis, was induced by TRAIL and LY294002, which are known to cause apoptosis [13], but not by the FU and hmUdR combination (Figure 3A). In addition, treatment with Quinolyl-valyl-*O*-methylaspartyl-[-2,6-difluorophenoxy]-methyl ketone (QVD), a pan-caspase inhibitor that blocks apoptosis [14], did not diminish the growth inhibition effect of FU and hmUdR as observed in either the WST-1 assay (Figure 3B) or the CyQUANT assay (data not shown). Next we determined changes in the levels of p62 [15] and LC3-II proteins [16], which are indicative of autophagy. Alterations in these proteins were not detected in cells treated with the FU and hmUdR combination (Figure 3C). Finally we used a necroptosis-specific inhibitor, necrostatin-1 (Nec-1) [17], and found that it did not reduce the growth inhibition effect of FU and hmUdR (Figure 3D and data not shown). Together these results demonstrate that treatment of HT-29 cells with the FU and hmUdR combination does not induce apoptosis, autophagy or necroptosis, and suggest that the combination of FU and hmUdR induces necrosis as a consequence of PARP1-dependent NAD depletion [18].

### Analysis of derivatives of FU and hmUdR for their synergistic activity

**Figure 4:**
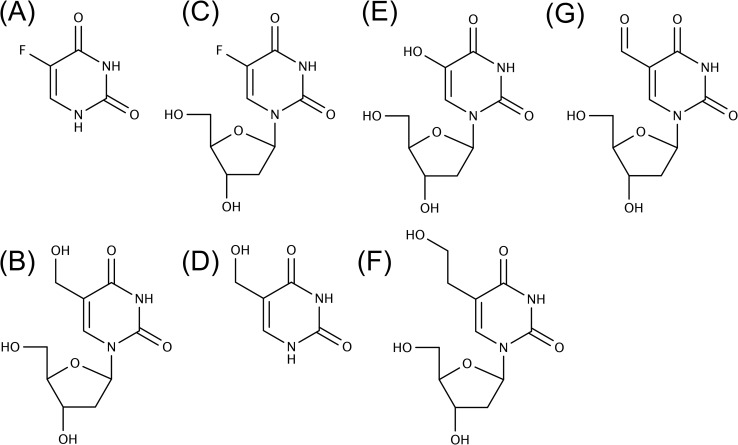
Chemical structures of base/nucleoside analogs tested in this study (A) FU. (B) hmUdR. (C) FUdR. (D) hmU. (E) hUdR. (F) heUdR. (G) foUdR.

**Figure 5:**
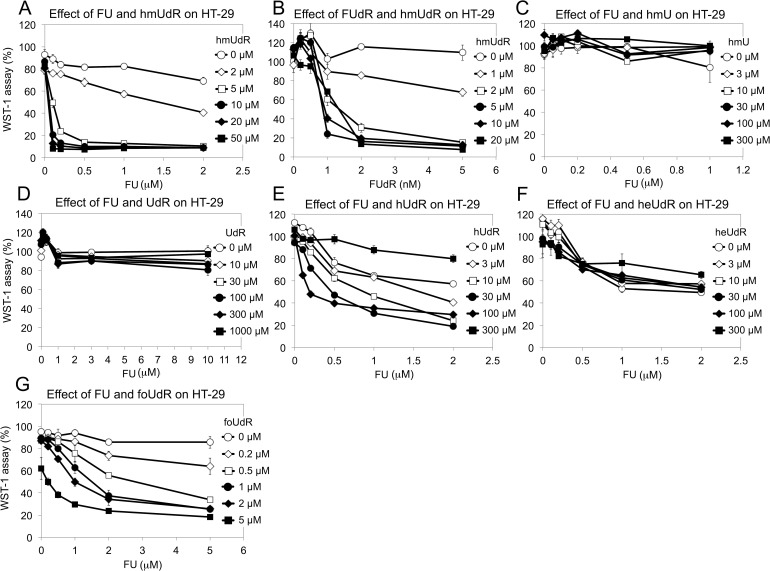
Effect of various drug combinations on the growth of HT-29 cells (A) FU and hmUdR. (B) 5-fluoro-2′-deoxyuridine (FUdR) and hmUdR. (C) FU and hmU. (D) FU and 2′-deoxyuridine (UdR). (E) 5-hydroxy-2′-deoxyuridine (hUdR) and FU. (F) 5-hydroxyethyl-2′-deoxyuridine (heUdR) and FU. (G) 5-formyl-2′-deoxyuridine (foUdR) and FU. HT-29 cells were treated with indicated compounds for 72 hours, and the cell proliferations were measured by WST-1 assay. Data are from triplicate experiments and plotted with standard deviations.

Since results that we obtained with the WST-1 assay correlated with the number of DNA single strand breaks and cytotoxicity generated by FU and hmUdR, we used this assay to determine the activity of several compounds that are structurally and/or functionally related to FU or hmUdR (Figure 4). When combined with hmUdR, the GI50 of HT-29 cells for FU was drastically decreased from 19 μM to less than 0.1 μM (Figure 5A). 5-Fluoro-2′-deoxyuridine (FUdR), a nucleoside derivative of FU with anti-cancer activity similar to FU [1], also acted synergistically with hmUdR, (Figure 5B). In contrast, 5-hydroxymethyluracil, a base derivative of hmUdR, did not significantly enhance FU activity (Figure 5C). Four derivatives of hmUdR, 2′-deoxyuridine (UdR), 5-hydroxy-2′-deoxyuridine (hUdR), 5-hydroxyethyl-2′-deoxyuridine (heUdR), and 5-formyl-2′-deoxyuridine (foUdR) were also evaluated. Both foUdR (Figure 5G) and, to a lesser extent, hUdR (Figure 5E) acted synergistically with FU. The activity of foUdR with FU was comparable to that of hmUdR. In contrast, neither UdR nor heUdR significantly enhanced FU activity (Figure 5D and F).

### Synergistic activity of FU and hmUdR in cancer but not normal cells

**Figure 6:**
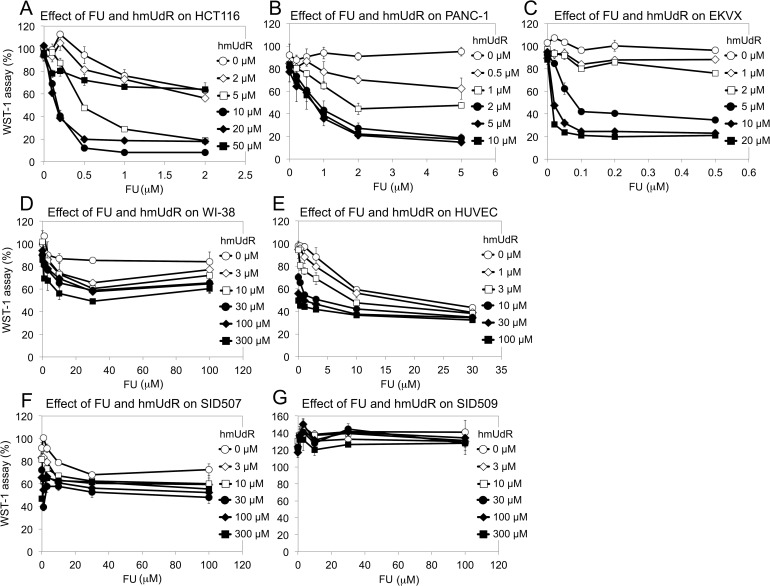
Effect of FU and hmUdR on the growth of various cells (A) HCT 116 (p53-proficient colorectal carcinoma). (B) PANC-1 (pancreatic cancer). (C) EKVX (non-small cell lung cancer). (D) A normal cell line, WI-38 (embryonic lung fibroblast). (E) Human umbilical vein endothelial cells (HUVEC). These cells were treated for 72 hours with increasing concentrations of FU and hmUdR, and their proliferations were measured by WST-1 assay. (F) SID507 (normal human colon cell line). (G) SID509 (normal human colon cell line). These normal colon cells were tested by the same procedures as above except that they were incubated with or without FU and hmUdR for 7 days. Data are from triplicate experiments and plotted with standard deviations.

Since hmUdR synergistically enhances the killing of p53 mutant colon cancer cells by FU, we asked whether this combination of nucleoside/base analogs has similar activity in other cancer cell lines and comparable non-malignant cell lines. First we examined another colorectal carcinoma cell line, HCT 116, that has wild type p53 but is defective in DNA mismatch repair. We obtained similar results to those of HT-29 cells except at the highest hmUdR concentration tested, 50 μM (Figure 6A). Nonetheless, it is evident that a combination of FU and up to 20 μM hmUdR synergistically inhibited the growth of colon cancer cell lines *in vitro* regardless of their p53 status. Cell lines derived from other tumor types were also tested for growth inhibition by FU and hmUdR. PANC-1 cells from pancreas and EKVX cells from lung also showed highly synergistic responses to these compounds at relatively low concentrations (Figure 6B and C). In contrast, comparable normal cell lines (WI-38 lung fibroblasts, Figure 6D; SID507 and SID509 normal human colon cell lines, Figure 6F and G) exhibited either no synergy with FU and hmUdR or a modest degree of synergy (human umbilical vein endothelial cells [HUVECs], Figure 6E).

To quantify the synergy of the FU and hmUdR in a more rigorous fashion, we calculated combination indexes for each cell line. The combination index method was developed to evaluate drug interaction, based on the multiple drug-effect equation of Chou-Talalay [19]. These indexes can be interpreted as follows: very strong synergism for < 0.1; strong synergism for 0.1-0.3; synergism for 0.3-0.7; moderate to slight synergism for 0.7-0.9; nearly additive for 0.9-1.1 [20]. As shown in Table 1, the combination indexes of the tumor cell lines were 0.11 or less at low concentrations of FU. In contrast, the HUVECs had a combination index of 0.34, and the combination indexes for the WI-38, SID507 and SID509 cell lines were not obtained because their growth inhibition did not reach 50%. Taken together, these findings reinforce the notion that the combination treatment of FU and hmUdR selectively impairs the viability of cancer cells compared with normal cells.

**Table 1:** Growth Inhibition and Combination Index of FU and hmUdR

	Growth Inhibition (%) with 1 μM FU + 10 μM hmUdR	Combination Index for GI50
Cancer cells		
HT-29 (colon)	89 ± 0.6	0.019
HCT 116 (colon)	92 ± 3.0	0.11
PANC-1 (pancreas)	59 ± 5.5	< 0.054 ^2^
EKVX (lung)	77 ± 0.2 ^1^	< 0.027 ^2^
Normal cells		
WI-38 (lung)	11 ± 5.8	ND ^3^
HUVEC (umbilical vein)	44 ± 5.2	0.34
SID507 (colon)	37 ± 4.5 ^4^	ND
SID509 (colon)	−30 ± 5.4 ^4^	ND

1 Treatment with 0.5 μM FU + 10 μM hmUdR.

2 GI50 of hmUdR was not determined but assumed as more than 300 μM.

3 Not determined.

4 Treatment with 3 μM FU + 10 μM hmUdR for 7 days.

## DISCUSSION

FU has been a mainstay of chemotherapy for colon cancer and other malignancies. Currently, it is frequently used in combination therapies with other genotoxic agents, such as oxaliplatin and irinotecan [2]. In this study, we report the novel and unexpected observation that the deoxyuridine analogs, hmUdR, hUdR and foUdR, synergistically enhance the sensitivity of a variety of cell lines derived from solid tumors but not cell lines from normal tissues to FU. Notably, this synergy was independent of p53 status and occurred in mismatch repair-defective HCT 116 cells [21] that also harbor a mutation in the thymidylate synthase gene that may confer some resistance to FU [22,23].

FU exerts pleiotropic effects on nucleic acid metabolism, disrupting RNA metabolism, nucleotide biosynthesis and DNA replication and repair. While our results do not exclude the possibility that the combination of FU and the deoxyuridine analogs synergistically inhibit RNA metabolism, the dramatic increase in DNA single strand breaks indicates that the combination of FU with one of the active deoxyuridine analogs is synergistically impacting the integrity of genomic DNA. In support of this, we observed that much lower concentrations of FUdR (5 nM versus 500 nM FU), which results in significantly more FU incorporation into DNA compared with FU [24], were required to synergistically inhibit cell proliferation and viability with hmUdR. Furthermore, while cells treated with the combination of FU and one of the deoxyuridine analogs accumulate a large number of DNA single strand breaks and arrest in S phase, the S phase arrest was alleviated by the addition of PARP inhibitors. Thus, it is unlikely that alterations in nucleotide pools resulting from inhibition of thymidylate synthase or other enzymes involved in nucleotide biosynthesis are responsible for the inhibition of DNA replicative synthesis by the combination of FU and one of the active deoxyuridine analogs. Instead, it is more likely that dNTP and ATP levels are reduced indirectly as a result of NAD depletion resulting from PARP1 activation by the single strand breaks.

Although PARP1 participates in many different aspects of DNA metabolism, it is a key player in the efficient repair of DNA single strand breaks, generating the signal, poly(ADP-ribose) that recruits single strand break repair proteins to the damage site [12]. Recently PARP inhibitors have been developed as cancer therapeutics because of their ability to cause replication-dependent DNA double strand breaks. These lesions cannot be repaired in cancers, such as hereditary forms of breast and ovarian cancer, that are defective in recombinational repair, resulting in cell death by apoptosis [25]. Conversely, DNA damaging agents such as DNA alkylating agents that generate large number of single strand breaks activate PARP1. This in turn induces a necrotic cell death as a consequence of NAD depletion that has been termed programmed necrosis [18,26]. Our results indicate that the combination of FU and hmUdR induces programmed necrosis since cell death is dependent on PARP activity, occurs in actively proliferating cells and is triggered by DNA damage. Interestingly, if PARP1-dependent necrosis is suppressed with a PARP inhibitor, the cells accumulate at G /M as a result of activation of an ATR/ATM-dependent checkpoint and then die by an as yet undefined mechanism.

It is likely that the single strand breaks observed in cells treated with FU and hmUdR result from their misincorporation during DNA replication followed by their removal by base excision repair [27-29]. Interestingly, hmUdR increases the incorporation of Ara-C, another pyrimidine analog inhibitor of DNA replication and nucleotide metabolism that is used primarily in the treatment of acute myeloid and acute lymphocytic anemia, to inhibit cell growth [10]. In contrast, hmUdR did not increase the incorporation of FU nor vice versa, indicating that a different mechanism underlies the synergistic activity of FU and hmUdR. It has been reported that the toxicity of FU correlates with thymine DNA glycosylase activity [29] whereas deficiency in 5-hydroxymethyluracil-DNA-glycosylase (SMUG1) activity confers resistance to hmUdR [30]. Furthermore, SMUG1 is also the major enzyme responsible for the removal of foU and hU [31], two of the deoxyuridine analogs that exhibited synergistic activity with FU. Further studies are needed to determine whether the substrate specificity and activity of SMUG1 with the deoxyuridine derivatives correlates with the ability of the deoxyuridine derivatives to act synergistically with FU. Since there was no increase in incorporation of modified nucleotides when cells were co-incubated with FU and hmUdR, it seems unlikely that the single strand breaks are generated simply as a consequence of exceeding the capacity of the steps following base removal in the base excision repair pathway. However, it is conceivable that, while alterations in nucleotide pools caused by FU and, possibly hmUdR, do not significantly impact replicative DNA synthesis, they may inhibit repair DNA synthesis. For example, the Km of Pol β for dNTP is significantly higher than that of Pol δ [32,33]. In this scenario, we suggest that the synergistic increase in single strand breaks generated in cells co-incubated with FU and hmUdR is caused by incomplete repair of misincorporated FU and hmUdR due to inhibition of repair synthesis. This hypothesis remains to be tested.

In summary, we have found that several deoxyuridine analogs synergistically enhance the cytotoxicity of both FU and FUdR, in cancer but not normal cells. Since both these drugs have been used extensively in the treatment of solid tumors, our results provide a rationale for the development of novel FU-based therapies that may be more effective both in terms of treating the tumors and in reducing toxicity to normal tissues and cells.

## MATERIALS AND METHODS

### Chemicals

QVD was obtained from R&D Systems. LY294002 and TRAIL were purchased from Cayman Chemical and PeproTech, respectively. Caffeine was obtained from USB. ABT-888 was purchased from Enzo Life Sciences. 5-formyl-2′-deoxyuridine was synthesized and purified as previously described [34]. All other chemicals were obtained from Sigma-Aldrich.

### Cell culture

HT-29 (derived from colorectal adenocarcinoma) and PANC-1 cells (derived from pancreatic carcinoma) were cultured in 4.5 g/l glucose-containing DMEM supplemented with 10% fetal bovine serum (FBS), 100 units/ml penicillin, 100 μg/ml streptomycin and 2 mM glutamine. HCT 116 cells (derived from colorectal carcinoma) were cultured in McCoy's 5A medium supplemented with 10% FBS, 100 units/ml penicillin, 100 μg/ml streptomycin and 2 mM glutamine. EKVX cells (derived from lung adenocarcinoma) were cultured in RPMI medium supplemented with 10% FBS, 100 units/ml penicillin, 100 μg/ml streptomycin and 2 mM glutamine. WI-38 cells (derived from normal lung fibroblast) were cultured in 4.5 g/l glucose-containing DMEM supplemented with 20% FBS, 100 units/ml penicillin, 100 μg/ml streptomycin and 2 mM glutamine, 1 mM pyruvate and 1× vitamin solution (Invitrogen). HUVECs were obtained from Genlantis and cultured in the endothelial cell growth medium supplied by Genlantis. All the cells were maintained in 5% CO_2_ at 37°C. SID507 and SID509 cells (untransformed colonocytes isolated from an individual with familial adenomatous polyposis by M. Clapper and obtained from the Cell Culture Facility at Fox Chase Cancer Center) were cultured in 4.5 g/l glucose-containing DMEM supplemented with 15% FBS, 100 units/ml penicillin, 100 g/ml streptomycin and 2 mM glutamine and 1 mM pyruvate.

### Colony formation assay

HT-29 cells were seeded at 6 × 10^4^ cells /well in 6-well plates, and on the next day, indicated compounds were added (0.5 μM for FU, 5 μM for hmUdR). After incubation for indicated time periods (0, 24, 48 or 72 h), cells were trypsinized, washed and replated into 6 cm dishes using appropriate dilutions and then incubated for 10 days without drugs. Colonies were stained with 0.25% methylene blue/30% ethanol, and counted. All assays were carried out in triplicate.

### Comet assay

HT-29 cells were seeded at 4 × 10^5^ cells /well in 6-well plates, and on the next day, indicated nucleosides and/or bases were added (0.5 μM for FU, 5 μM for hmUdR). After incubation for indicated time periods (12-48 h), the cells were trypsinized and washed in PBS. For time course experiments, cells harvested at each time point were stored in 10% DMSO/40% DMEM/50% FBS at −80°C until slide processing. Approximately 5,000 cells were spread in 0.9% low-melting point agarose/PBS on CometSlide (Trevigen), and chilled at 4°C in the dark for 20 min.

For alkaline comet assay, slides were soaked in precooled lysis buffer containing 2.5 M NaCl/100 mM EDTA/10 mM Tris/1% sarkosyl/1% Triton X-100 at 4 °C for 45 min, followed by soaking in precooled 300 mM NaOH/1 mM EDTA at 4°C for 45 min. Subsequently, slides were electrophoresed in 300 mM NaOH/1 mM EDTA at 1.4 V/cm for 20 min at 4°C, washed in 70% ethanol for 5 min, and allowed to dry in the dark. Cellular DNA was stained with 1× SYBR Green I (Molecular Probes) 30 min before analysis with a fluorescence microscope. Alkaline comet assays were performed in triplicate and more than 30 comets for each condition were photographed at the Light Microscope Facility at Fox Chase Cancer Center, and analyzed by CometScore software (TriTek).

For neutral comet assay, slides were soaked in precooled lysis buffer at 4°C for 30 min, followed by washing in precooled 1 x TBE buffer (90 mM Tris-borate, pH8.3, 2 mM EDTA). Slides were electrophoresed in 1 x TBE buffer at 2 V/cm for 20 min at 4°C, rinsed in deionized water, washed in 70% ethanol for 5 min, and allowed to dry in the dark. Subsequently, slides were processed as above for DNA staining and comet analyses. Neutral comet assays were conducted in duplicate, in each of which more than 60 comets for each condition were analyzed.

### Quantitation of FU and hmUdR incorporated into cellular DNA

[6-^3^H]-FU (18 Ci/mmol) and of [^3^H]-hmUdR (10 Ci/mmole) were purchased from Moravek Biochemicals. HT-29 cells were seeded at 5 × 10^5^ cells /well (for treatment with one compound only) or 10 × 10^5^ cells / well (for treatment with FU and hmUdR) in 6-well plates one day before drug addition. For FU quantitation, 0.5 μM FU and 5 μCi/well of [6-^3^H]-FU were added to the medium together with or without 5 μM nonradioactive hmUdR in triplicate. For hmUdR quantitation, 5 μM hmUdR and 1 Ci/well of [^3^H]-hmUdR were added to the medium together with or without 0.5 μM nonradioactive FU in triplicate. At 24 or 48 h after drug addition, cells were washed with PBS and their DNA was recovered with Trizol (Invitrogen) according to the manufacturer's instruction. Subsequently the recovered DNA was quantitated by 260 nm absorbance, and its radioactivity was measured by liquid scintillation counting.

### Synchronization of cultured cells at the G_1_/S boundary

HT-29 cells that were seeded at 2 × 10^6^ cells /plate in 10 cm dishes and incubated with 20 ng/ml nocodazole for 18 h. After washing with PBS, 1 μg/ml aphidicolin and, where indicated, 0.5 μM FU and 5 μM hmUdR were added for 12 h. The synchronized cells were washed with PBS prior to the addition of fresh medium containing the indicated nucleosides and/or bases.

### Cell cycle analysis

Cells grown in 10 cm dishes were trypsinized, spun down and suspended in 10 ml PBS containing 0.5% FBS. After centrifugation, the cells were resuspended in 0.5 ml PBS/0.5% FBS, and fixed in 5 ml 70% ethanol at −20°C. After centrifugation and washing with 10 ml PBS/0.5% FBS, the cells were suspended in 1.5 ml PBS/0.5% FBS containing 10 μg/ml propidium iodide and 50 μg/ml RNase A, and incubated at 37°C for 30 min. Cell cycle distribution was analyzed with a FACScan flow analyzer (Becton Dickinson).

### Time-lapse image acquisition

HT-29 cells were infected with a retroviral vector for expression of GFP-fused histone H2B. HT-29 cells expressing GFP-H2B were seeded at 2 × 10^5^ cells /well in 6-well plates. On the following day, drug treatments were initiated and cell proliferation was monitored by time-lapse microscopy. Image acquisition was done at the Light Microscope Facility at Fox Chase Cancer Center using phase-contrast and GFP-specific fluorescence microscopy (Nikon TE2000S) controlled by Metamorph (Molecular Devices). Images were captured at a rate of one frame per 15 minutes for 60 hours, in which cells were kept at 370C. Images were captured from 10 areas per well. The number of cell divisions that occurred in each area was counted for the first 24 h and the second 24 h periods.

### Immunoblotting

For detection of PARP1 cleavage and autophagy-related proteins, the HT-29 cells treated as indicated were washed with PBS and resuspended in 40 mM HEPES-KOH, pH7.5/500 mM NaCl/10% glycerol/0.1% NP-40/Protease Inhibitor Cocktail III for mammalian cells (Research Products International Corp). After 10 min on ice, cells were scraped and centrifuged at 16,000 × g for 10 min at 4°C. The supernatant was recovered. This whole cell extract (50 μg protein) was subjected to SDS-containing polyacrylamide gel electrophoresis, and transferred to Immobilon-P membrane (Millipore). For detection of poly(ADP-ribose), the nuclear pellet was recovered after removing the whole cell extract as prepared above except that the lysis buffer was supplemented with 50 μM ethacridine, an inhibitor of poly(ADP-ribose) glycohydrolase. 10 μg protein of the nuclear pellet was subjected to gel electrophoresis and transfer to membrane as described above. Primary antibodies used in this study were anti-PARP1 monoclonal mouse antibody (Trevigen), anti-p62 polyclonal rabbit antibody (Santa Cruz Biotechnology), anti-LC3 polyclonal rabbit antibody (Novus Biologicals), anti-β-actin monoclonal mouse antibody (Sigma), and anti-PCNA monoclonal antibody (PC10; Santa Cruz Biotechnology), anti-poly(ADP-ribose) mouse monoclonal antibody (Tulip Biolabs). As secondary antibodies, either IRDye800CW-conjugated anti-mouse IgG antibody, IRDye700-conjugated anti-rabbit IgG antibody (both from LI-COR Biotechnology) or horseradish peroxidase-conjugated anti-mouse IgG antibody (Bio-Rad Laboratories) was used. Immunoblot signals were detected either by Odyssey Imaging System (LI-COR Biotechnology) or by exposure of X-ray films to the membrane soaked in ECL reagent (GE Healthcare).

### Cell growth/viability assays

In the WST-1 assay measuring cell growth and viability, cells were seeded in 96-well plates at the following densities: 10,000 cells/well for HT-29; 2,500 cells /well for HCT 116; 1,000 cells/well for PANC-1; 5,000 cells/well for EKVX; 3,000 cells/well for WI-38; 3,000 cells/well for SID-507 and SID-509; 2,000 cells/well for HUVECs. Indicated concentrations of drugs were added to wells one day after seeding. After three days incubation with the indicated nucleosides and/or bases (except for SID-507 and -509 cells which were incubated for seven days), 5 μl WST-1 reagent (Roche) was added to each well, and plates were further incubated at 37°C for 3 h. Cell proliferation was quantitated by measuring 450 nm absorbance and 600 nm as a background. All assays were performed in triplicate.

Cell proliferation assays measuring genomic DNA were carried out using the CyQUANT kit (Invitrogen). In these experiments, the cells after drug treatments were replated to grow in the absence of the drugs for six days, and their nucleic acids was quantitated by CyQUANT assay. These assays were also conducted in triplicate.

### Determination of cellular NAD levels

Extracts of cells treated as indicated were prepared as described by Zong et al. [26]. Protein concentrations were measured with the BCA protein assay reagent (Pierce). NAD concentrations were determined with NAD+/NADH cell-based assay kit (Cayman Chemical) and normalized using protein concentration.

### Evaluation of drug interactions

Parameters of an isobologram for 50% growth inhibition (GI50) were calculated from data obtained from simultaneous treatment with the two drugs by assuming that the isobole fits to a hyperbolic curve. The minimal combination index [20] for each cell line was obtained from the isobologram parameters.

## SUPPLEMENTARY MATERIALS, FIGURES


