# Supplementary material for: Synergistic enhancement of 5-fluorouracil cytotoxicity by deoxyuridine analogs in cancer cells
Source: Oncoscience. 2015 Feb 9;2(3):272–84. doi: 10.18632/oncoscience.125 (PMC4394133; doi:10.18632/oncoscience.125)
Supplement: Supplementary file 1 [file oncoscience-02-0272-s001.pdf]

Synergistic enhancement of 5-fluorouracil cytotoxicity by deoxyuridine analogs in cancer cells

Supplementary Material

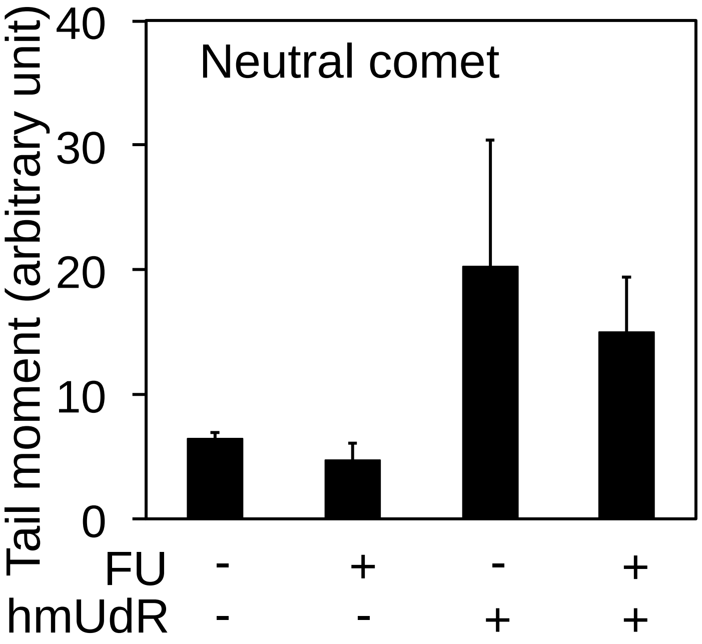

Supplementary Figure 1: Neutral comet assays for detection of double-strand breaks (DSBs) in HT-29 cells treated for 48 h with indicated combinations of 0.5 μM FU and 5 μM hmUdR.

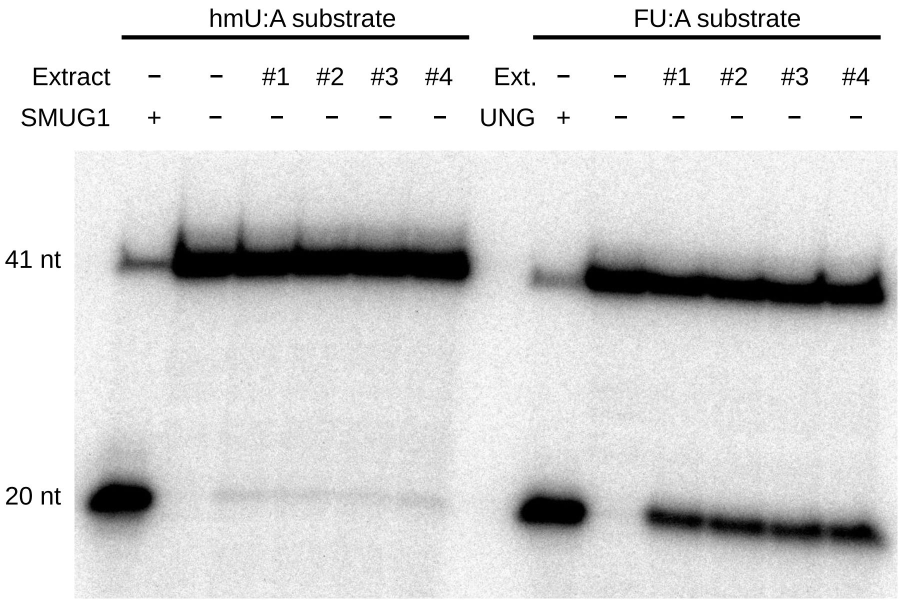

Supplementary Figure 2: Base excision activity of HT-29 cell extract. 124 fmol of double-stranded oligonucleotide containing an hmU:A or FU:A pair was incubated at 37°C for 1 h with either an indicated recombinant enzyme or 10 μg protein of the following whole cell extracts: #1 from untreated cells; #2 from the cells treated with 0.5 μM FU for 48 h; #3 from the cells treated with 5 μM hmUdR for 48 h; #4 from the cells treated with 0.5 μM FU and 5 μM hmUdR for 48 h. After incubation, the oligonucleotides were treated with 1 N NaOH at 90°C for 5 min, neutralized with 1 M acetic acid, and subjected to 20% sequencing gel electrophoresis. 41 nt, uncleaved substrate; 20 nt, product cleaved at hmU or FU site after damage-specific DNA glycosylase reaction.

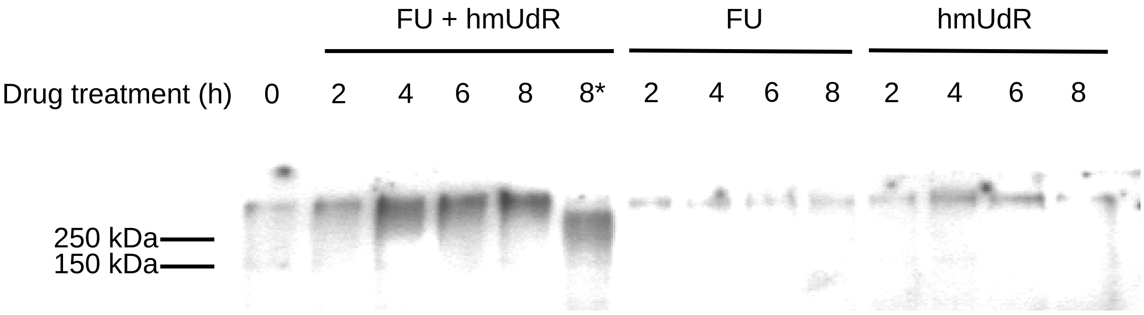

Supplementary Figure 3: Immunoblot detection of poly(ADP-ribose). HT-29 cells were treated with indicated drugs for indicated time periods and their nuclear pellets were prepared as described in Materials and Methods. All samples except the sample indicated with an asterisk were processed in the lysis buffer supplemented with 50 μM ethacridine, a poly(ADP-ribose) glycohydrolase inhibitor.
